# Accomplishing the genotype-specific serodiagnosis of single and dual *Trypanosoma cruzi* infections by flow cytometry Chagas-Flow ATE-IgG2a

**DOI:** 10.1371/journal.pntd.0006140

**Published:** 2018-02-20

**Authors:** Glaucia Diniz Alessio, Fernanda Fortes de Araújo, Policarpo Ademar Sales Júnior, Matheus de Souza Gomes, Laurence Rodrigues do Amaral, Marcelo Antônio Pascoal Xavier, Andréa Teixeira-Carvalho, Marta de Lana, Olindo Assis Martins-Filho

**Affiliations:** 1 Laboratório de Doença de Chagas, Núcleo de Pesquisas em Ciências Biológicas (NUPEB), Instituto de Ciências Exatas e Biológicas (ICEB), Universidade Federal de Ouro Preto (UFOP), Ouro Preto, MG, Brazil; 2 Grupo Integrado de Pesquisas em Biomarcadores, Instituto René Rachou (FIOCRUZ-Minas), Belo Horizonte, MG, Brazil; 3 Programa de Pós-graduação em Sanidade e Produção Animal nos Trópicos, Universidade de Uberaba, Uberaba, Brazil; 4 Grupo de Genômica Funcional e Proteômica de *Leishmania spp* e *Trypanosoma cruzi*, Instituto René Rachou (FIOCRUZ-Minas), Belo Horizonte, MG, Brazil; 5 Laboratório de Bioinformática e Análises Moleculares, Universidade Federal de Uberlândia, INGEB/FACOM, Campus Patos de Minas, Patos de Minas, MG, Brazil; 6 Grupo de Pesquisas Clínicas e Políticas Públicas em Doenças Infecciosas e Parasitárias, Instituto René Rachou (FIOCRUZ-Minas), Belo Horizonte, MG, Brazil; Instituto de Ciências Biológicas, Universidade Federal de Minas Gerais, BRAZIL

## Abstract

The methods currently available for genotype-specific diagnosis of *T*. *cruzi* infection still present relevant limitations, especially to identify mixed infection. In the present investigation, we have evaluated the performance of Chagas-Flow ATE-IgG2a test for early and late differential diagnosis of single and dual genotype-specific *T*. *cruzi* infections. Serum samples from Swiss mice at early and late stages of *T*. *cruzi* infection were assayed in parallel batches for genotype-specific diagnosis of single (TcI, TcVI or TcII) and dual (TcI+TcVI, TcVI+TcII or TcII+TcI) infections. The intrinsic reactivity to TcI, TcVI and TcII target antigens, including amastigote (AI/AVI/AII), trypomastigote-(TI/TVI/TII) and epimastigote (EI/EVI/EII), at specific reverse of serum dilutions (500 to 64,000), was employed to provide reliable decision-trees for “early” *vs* “late”, “single *vs* “dual” and “genotype-specific” serology. The results demonstrated that selective set of attributes “EII 500/EI 2,000/AII 500” were able to provide high-quality accuracy (81%) to segregate early and late stages of *T*. *cruzi* infection. The sets “TI 2,000/AI 1,000/EII 1,000” and “TI 8,000/AII 32,000” presented expressive scores to discriminate single from dual *T*. *cruzi* infections at early (85%) and late stages (84%), respectively. Moreover, the attributes “TI 4,000/TVI 500/TII 1,000”, “TI 16,000/EI 2,000/EII 2,000/AI 500/TVI 500” showed good performance for genotype-specific diagnosis at early stage of single (72%) and dual (80%) *T*. *cruzi* infections, respectively. In addition, the attributes “TI 4,000/AII 1,000/EVI 1,000”, “TI 64,000/AVI 500/AI 2,000/AII 1,000/EII 4,000” showed moderate performance for genotype-specific diagnosis at late stage of single (69%) and dual (76%) *T*. *cruzi* infections, respectively. The sets of decision-trees were assembled to construct a sequential algorithm with expressive accuracy (81%) for serological diagnosis of *T*. *cruzi* infection. These findings engender new perspectives for the application of Chagas-Flow ATE-IgG2a method for genotype-specific diagnosis in humans, with relevant contributions for epidemiological surveys as well as clinical and post-therapeutic monitoring of Chagas disease.

## Author summary

*Trypanosoma cruzi* shows great genetic diversity, and was subdivided into six DTUs (Discrete Typing Units), named TcI-TcVI. This genetic and biological variability, coupled with natural reinfection of hosts, may play an important role in the clinical and epidemiological features of the disease. Furthermore, hosts infected with different *T*. *cruzi* genotypes demonstrated distinct therapeutic response. Thus, the development of new methods for genotype-specific diagnosis of *T*. *cruzi* infection is very important for clinical and epidemiological studies and for post-therapeutic monitoring of patients treated. Biochemical and molecular methods are used for the purpose, however, these techniques have methodological limitations. In addition, the standardized serological methods for genotype-specific diagnosis of Chagas disease present antigenic limitations and also do not evaluate the reactivity of serum samples from mixed infections. In order to overcome these challenges, our group developed the Chagas-Flow ATE-IgG2a technique with good performance for universal and genotype-specific diagnosis of single *T*. *cruzi* infection in the chronic phase. Based on our previous results, in the present investigation, we evaluated the applicability of Chagas-Flow ATE-IgG2a in the genotype-specific diagnosis at early and late stages for single and dual *T*. *cruzi* infections.

## Introduction

Chagas disease, caused by the protozoan parasite *Trypanosoma cruzi*, is an endemic antropozoonosis in Latin America that affects about 6–7 million people worldwide [1]. The disease progresses from an acute phase, with high parasitemia and unspecific symptoms, to a lifelong chronic infection, with subpatent parasitemia and asymptomatic clinical status in most patients, or cardiovascular/gastrointestinal involvement in a minor proportion of infected individuals [2–4].

It has been proposed that besides the influence of genetic background of the infected subjects, the genetic variability of *T*. *cruzi* contribute to the clinical course of Chagas disease, defined by the particular tropism of *T*. *cruzi* genotypes to distinct host tissues [5, 6]. The current classification consensus proposes six genetic groups or Discrete Typing Units (DTUs) of *T*. *cruzi* stocks, referred as TcI to TcVI, based on different molecular markers and biological features [7]. In general, it has been considered that vertebrate and invertebrate hosts may present mixed infections with multiple *T*. *cruzi* genotypes that can lead to changes in the parasite biological properties and also the clinical course of Chagas disease [5, 8–19]. The resulting characteristics of single infections and mixed infections can also influence the outcome of specific etiological treatment [14, 15].

In this context, the identification of single and mixed *T*. *cruzi* infections upon the diagnosis is of extreme importance, as it can have direct impact in prognosis of disease morbidity and response to chemotherapy. Usually, the diagnosis of acute Chagas disease is carried out mostly by direct parasitological methods (fresh blood examination, thick smear and dry smear) while in the chronic phase is usually diagnosed by serological methods [20]. Nowadays, molecular methods have been widely used for genotype-specific diagnosis of single and mixed *T*. *cruzi* infection during acute and chronic infections [21]. In general, the molecular approaches require the use of several genetic markers to distinguish the *T*. *cruzi* genotype [22–24]. Moreover, in cases of mixed infections, the molecular tests may identify distinct *T*. *cruzi* genotypes, depending on: i) the biological sample tested (distinct tissue tropism); ii) the need for parasite isolation (clonal selection) and iii) the requirement of further expansion of parasites by *in vitro* or *in vivo* growth to obtain large amounts of genetic material (low sensitivity) [5, 6, 13, 17–19, 22, 25].

Aiming at developing a more reliable method for genotype-specific diagnosis of *T*. *cruzi* infections, with simple system of high performance, innovative serological methods have been proposed [26–30]. In general, most serological methods already described for genotypic-specific diagnosis of *T*. *cruzi* infection have employed polymorphic B-cell epitopes identified amongst distinct *T*. *cruzi* strains. Bhattacharyya *et al*. (2010, 2014 and 2015) [27, 29, 30], working with TSSA (trypomastigote small surface antigen), representative of the TcMUC III gene family, have demonstrated lineage-specific epitopes within TcII-VI with implications for sero-epidemiological studies [27, 29, 30]. Moreover, Mendes *et al*. (2013) [28] have described specific *T*. *cruzi* peptides, derived from RNA binding proteins, RNA polymerase III and GTPase activating protein, with high-precision ability to discriminate the experimental infections with distinct *T*. *cruzi* genotypes using ELISA or affinity-ELISA. Despite the great advance to overcome the limitations of molecular methods, some of these innovative methods have been test only with single infections and were not able to identify all *T*. *cruzi* genotypes. Recently, the Chagas-Flow ATE-IgG2a methodology, originally proposed by Alessio *et al*. (2014) [31] was optimized for genotype-specific diagnosis of single *T*. *cruzi* chronic infection, with high-quality performance to discriminate TcI and TcII genotypes [32].

In the present study, the performance of genotype-specific Chagas-Flow ATE-IgG2a [32] was further evaluated for early and late differential diagnosis of single and dual *T*. *cruzi* infections.

## Methods

### Ethics statement

All procedures involving experimental animals were carried out in compliance with the guidelines issued by National Council for Control of Animal Experimentation for ethical conduct in use of animals in research. The study protocol was approved by the Ethics Committee on Animal Experimentation of the Universidade Federal de Ouro Preto (Protocol approval numbers #2013/48 from December, 6^th^, 2013 for the experimental infection and collected blood).

### Standard *Trypanosoma cruzi* genotypes stocks

Three standard *T*. *cruzi* strains, representative of the major genotypes associated with the domestic cycle of Chagas disease in Brazil, were selected for this study, including: Colombiana strain, “COL” (TcI) [33], CL strain (TcVI) [34] and Y strain (TcII) [35]. These *T*. *cruzi* stocks were used for the experimental infection and also as target antigens for Chagas-Flow ATE-IgG2a. All isolates were acquired from the *T*. *cruzi* cryobank at Grupo de Genômica Funcional e Proteômica de *Leishmania spp* e *Trypanosoma cruzi*, Instituto René Rachou (IRR-FIOCRUZ-Minas) and maintained by consecutive *in vivo* passages in Swiss mice to prepare inoculums for experimental infection and *in vitro* growth in tissue and axenic cultures to obtain target antigens for Chagas-Flow ATE-IgG2a.

### Experimental infections with distinct *T*. *cruzi* genotypes

Female Swiss mice (28–30 days old) were obtained from the Animal Science Center at Universidade Federal de Ouro Preto (UFOP), MG, Brazil, and maintained in a temperature-controlled room with access to water and food *ad libitum*.

For single *T*. *cruzi* infections, animals were intraperitoneally inoculated with 50 blood trypomastigotes of each *T*. *cruzi* strains and allocated into three subgroups referred as “TcI/COL”, “TcVI/CL” or “TcII/Y”. For dual *T*. *cruzi* infections animals were intraperitoneally inoculated with 25 blood trypomastigotes of each *T*. *cruzi* strains and subdivided into three subgroups referred as “TcI/COL+TcVI/CL”, “TcVI/CL+TcII/Y” or “TcII/Y+TcI/COL”. All infections were confirmed with parasitemia using fresh blood examination performed either at day 7, 10 or 15 post-infection. A group of non-infected mice (NI, n = 10) were included as a “control”.

Blood samples were collected without anticoagulant from each mouse by ocular plexus puncture at distinct time points after infection, referred as “early stage” (30 days post-infection) and “late stage” (90 and 180 days post-infection). Serum samples were prepared from whole blood, inactivated at 56°C, 30 min and stored at -20°C until use. According to the animal mortality during the experimental timeline, the total number of animals included in each group was: i) “early stage” of single infection: “TcI/COL” (n = 16), “TcVI/CL” (n = 15) and “TcII/Y” (n = 19); ii) “late stage” of single infection: “TcI/COL” (n = 29), “TcVI/CL” (n = 29) and “TcII/Y” (n = 35); iii) “early stage” of dual infection:“TcI/COL+TcVI/CL” (n = 16), “TcVI/CL+TcII/Y” (n = 24), “TcII/Y+TcI/COL” (n = 20) and iv) “late stage” of dual infection:“TcI/COL+TcVI/CL” (n = 28), “TcVI/CL+TcII/Y” (n = 43), “TcII/Y+TcI/COL” (n = 36).

### Preparation of target antigens for the Chagas-Flow ATE-IgG2a

The Amastigote/Trypomastigote/Epimastigote (ATE) target antigens were obtained as described previously by Alessio *et al*. (2014) [31]. Briefly, enriched live Trypomastigotes (“T”) and Amastigote (“A”) preparations of TcI, TcVI and TcII *T*. *cruzi* genotypes were harvested from supernatant of desynchronized *in vitro* tissue cultures after 4–6 days and 8–15 days post-inoculation, respectively. Epimastigote (“E”) forms of TcI, TcVI and TcII *T*. *cruzi* genotypes were obtained at log-phase growth in axenic culture and pre-fixed with FACS fix solution (10.0 g of paraformaldehyde, 10.2 g of sodium cacodylate and 6.65 g of sodium chloride/l, pH 7.2; Sigma Aldrich, St Louis, MO, USA).

Live “A/T” and pre-fixed “E” were labeled with fluorescein isothiocyanate/FITC (Sigma Aldrich, St Louis, MO, USA) as described previously by Alessio *et al*. (2014) [31]. In summary, “A/T” and “E” suspensions (1×10^7^/ml) were stained with FITC (100μg/mL for TcI/COL and 200μg/mL for TcVI/CL and TcII/Y) for 30 min at 37°C. After staining, “A/T” mix were re-incubated at 37°C for 60 min for differential quenching of FITC and “E” re-incubated at 4°C overnight as described previously by Alessio *et al*., (2014) [31]. Following, FITC-labeled ATE target antigens were mixed and monitored by flow cytometry to obtain a proportional suspension (33% “A”, 33% “T” and 33% “E”). Representative flow cytometric dot plot distribution of differential FITC labeling of target antigens are provided in the S1 Fig.

### TcI/TcVI/TcII genotype-specific Chagas-Flow ATE-IgG2a

The Chagas-Flow ATE-IgG2a methodology was carried out as described previously by Alessio *et al*. (2017) [32] and the samples were tested in a blind study design for each genotype-specific platforms. Aliquots of 50μL of pre-diluted (1:500 to 1:64,000 final concentration) 0.22μm-filtered serum samples were incubated in the presence of 50μL of “A/T/E” target antigens at 37°C for 30 min using parallel batches of TcI, TcVI and TcII genotype-specific platforms in U-bottom 96-well plates. After incubation, “A/T/E” target antigens were washed twice with phosphate-buffered-saline supplemented with 10% fetal bovine serum (PBS-10%FBS). The “A/T/E” target antigens were re-incubated with 50μL of pre-diluted (1:3,000) biotin-conjugated anti-mouse IgG2a (BD Bioscience, San Jose, CA, USA) plus 20μL of pre-diluted (1:800) streptavidin phycoerytrin–SAPE (BD Bioscience, San Jose, CA, USA) at 37°C for 30 min. “A/T/E” target antigens were washed twice with PBS-10%FBS, fixed with 200μL of FACS fixing solution and store at 4°C for 30 min and up to 24 hours prior flow cytometric acquisition. Second step reagents control (anti-mouse IgG2a-biotin+SAPE) was included in all experiments to monitor unspecific bindings.

Flow cytometric measurements were performed on a FACSCan flow cytometer (BD Bioscience, San Diego, CA, USA) using the CellQuest software package for data acquisition and storage. Appropriate instrument settings were used on log scale (Forward Scatter-FSC = E00, Side Scatter-SSC = 427, threshold = 400; FL1 = 620 and FL2 = 500). A total of 10,000 events were acquired per each tested sample. The FlowJo software version 10.1 (TreeStar, San Diego, CA, USA) was used for data analysis. Serum reactivity to “A/T/E” target antigens of each *T*. *cruzi* genotype was determined as follows: target antigens were selected based on their differential FITC-label characteristic and the analysis of the percentage of positive fluorescent parasites (PPFP) calculated on FL2 (α-IgG2a-biotin/SAPE) one-dimensional histograms upon the setting of positivity limit (PPFP<2%) on the internal control. An overview of the Chagas-Flow ATE-IgG2a experimental procedure is provided in S1 Fig.

### Statistical analysis and data mining for selecting sets of attributes

The R-project software, Version 3.0.1, was used to identify for each target antigen (“A”, “T” and “E”) the higher modular distance between the mean reactivity (PPFP) along the titration curves (1:500 a 1:64000) and identify the pairs of attributes (“target antigen and serum dilution”) for differential diagnosis “early” *vs* “late”, “single” *vs* dual and “genotype-specific” serology.

The GraphPad Prism software, Version 5.0 (San Diego, CA, USA) was used to perform the non-parametric Kruskal–Wallis test followed by the Dunns' post-test for multiple comparisons to select the “top” pairs of attributes (“target antigen and serum dilution”) with the most outstanding significant differences (p ≤ 0.05) of reactivity to target antigens amongst subgroups.

The MedCalc software package 7.3 (Ostend, Belgium) was used to obtain the Receiver Operating-Characteristic features and the Microsoft Excel 2010 employed to calculate the global median or global mean values as putative cut-off edges to define set of attributes (“target antigen and serum dilution/ cut-off”) to segregate the reactivity to target antigens amongst subgroups.

The WEKA software, version 3.6.11 (University of Waikato, New Zealand) was used to construct the decision trees based on the pre-selected set of attributes (target-antigen/serum dilution/cut-off) to create algorithms (root and branch) for differential diagnosis “early” *vs* “late”, “single” *vs* dual and “genotype-specific” serology. The global accuracy and the “leave-one-out-cross-validation” (LOOCV) values were employed as performance indices. The LOOCV, also referred as “rotation estimation” is a mathematical validation model for generalizing the results of a given statistical analysis to an independent data set. This strategy is usually applied in settings where the major aim is to estimate how accurate the predictive model will behave when applied in other studies.

The Microsoft Excel software was used for (i) radar charts assemblage and the (ii) reactivity board drawings. (i) The radar charts, also referred as “spider diagram” or “polar coordinate” is a graphical method to display multivariate data using a two-dimensional format with quantitative variables represented on axes starting from the same point, giving the plot a “flower-like” appearance, where the area of each spokes reflects the quantitative magnitude of each variable. (ii) The reactivity board summarizes the results of individual samples and gives an overall profile of the serological reactivity categorized by stage (early or late), type (single or dual) and genotype (TcI, TcVI and TcII). The GraphPad Prism package was used to create symbol&line graphs as well as box plots, and the Microsoft PowerPoint software was employed to draw the decision tree and table layouts.

## Results

### Panoramic overview of Chagas-Flow ATE-IgG2a reactivity during early and late experimental *T*. *cruzi* infections

**Fig 1:**
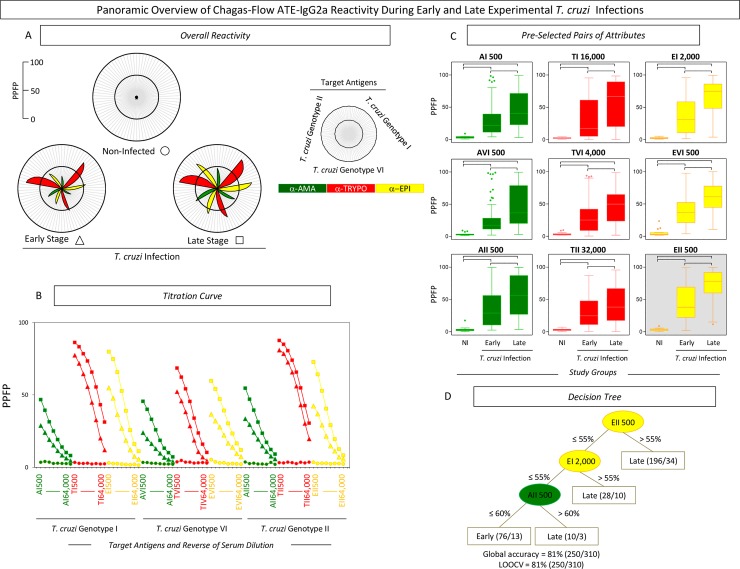
Chagas-Flow ATE-IgG2a performance to discriminate serological reactivity during early and late *T*. *cruzi* infections. (A) Overall reactivity profile of sera samples from early (n = 110) and late (n = 200) *T*. *cruzi* infections and non-infected hosts (n = 10). The IgG2a reactivity was evaluated along the titration curve with each target-antigens amastigote (AMA = green), trypomastigote (TRYPO = red) and epimastigote (EPI = yellow) from TcI, TcVI and TcII genotypes of *T*. *cruzi*. The results are displayed in radar charts and expressed as mean of percentage of positive fluorescent parasites (PPFP). (B) Titration curves were assessed at eight serum dilutions (1:500 to 1:64,000) using target-antigens AI, AVI and AII; TI, TVI and TII along with EI, EVI and EI at early (triangle) and late (square) stages of *T*. *cruzi* infection as compared to non-infected hosts (circle). (C) The reactivity amongst groups was compared for pre-selected pairs of attributes, including: AI 500; AVI 500; AII 500; TI 16,000; TVI 4,000; TII 32,000; EI 2,000; EVI 500 and EII 500. The results are expressed as median PPFP values in box plot format with the outliers underscored by shaded dots. Comparative analyses were performed by the Kruskal-Wallis/Dunn’s post test for multi-group comparisons. Significant differences were considered at p<0.05 and highlighted by connecting lines. The light gray background underscores the pair of attributes (“target antigen and serum dilution”) with the higher performance to discriminate early and late *T*. *cruzi* infections. (D) Decision trees were constructed using sets of attributes (“target-antigen and serum dilution/cut-off”) to create algorithms (root and branches) to classify individual samples at early and late *T*. *cruzi* infections. Global accuracy and leave-one-out-cross-validation-LOOCV are provided in the Figure.

The overall profile of Chagas-Flow ATE-IgG2a reactivity observed at early and late *T*. *cruzi* infections is presented in the Fig 1.

An overall profile of IgG2a reactivity to distinct target-antigen (“A”, “T” and “E”) of TcI, TcVI and TcII *T*. *cruzi* genotypes was first assessed along the titration curves using radar chart compilations of PPFP mean values. This panoramic snapshot provided evidences of differential reactivity during early and late *T*. *cruzi* infections as compared to non-infected hosts (Fig 1A and 1B). The results demonstrated persistent high reactivity to “T” at early and late stages of infection. Conversely, higher reactivity to “A” and “E” was evident during late infection (Fig 1A and 1B).

Comparative analysis, based on the modular distance between the mean reactivity (PPFP) along the titration curves and on box plot charts of selected target antigens, allowed the selection of pairs of attributes (“target antigen and serum dilution”) with the most promising performance to distinguish early and late *T*. *cruzi* infections (Fig 1C). The pairs of attributes “AI 500”; “TI 16,000”; “EI 2,000”; “AVI 500”; “TVI 4,000”; “EVI 500”; “AII 500”; “TII 32,000” and “EII 500” were pre-selected based on their ability to distinguish reactivity between early and late infections. The pair “EII 500” presented the “top” modular distance to discriminate the median reactivity at early and late infections (Fig 1C—gray background).

The ROC curve analyses were used to define specific cut-off edges for each pre-selected pairs of attributes and subsidize the construction of the most accurate decision tree to segregate the IgG2a reactivity to discriminate, at individual level, early and late infections.

Decision tree algorithm proposed the use of a sets of attributes (“target-antigen and serum dilution/cut-off”), including “EII 500/55%” (root), “EI 2,000/55%” (first branch) and “AII 500/60%” (second branch) to classify individual samples from early *vs* late infections, with elevated global accuracy (81.0%, LOOCV = 81.0%) (Fig 1D).

### Sets of attributes for differential diagnosis of single and dual at early and late *T*. *cruzi* infections

**Fig 2:**
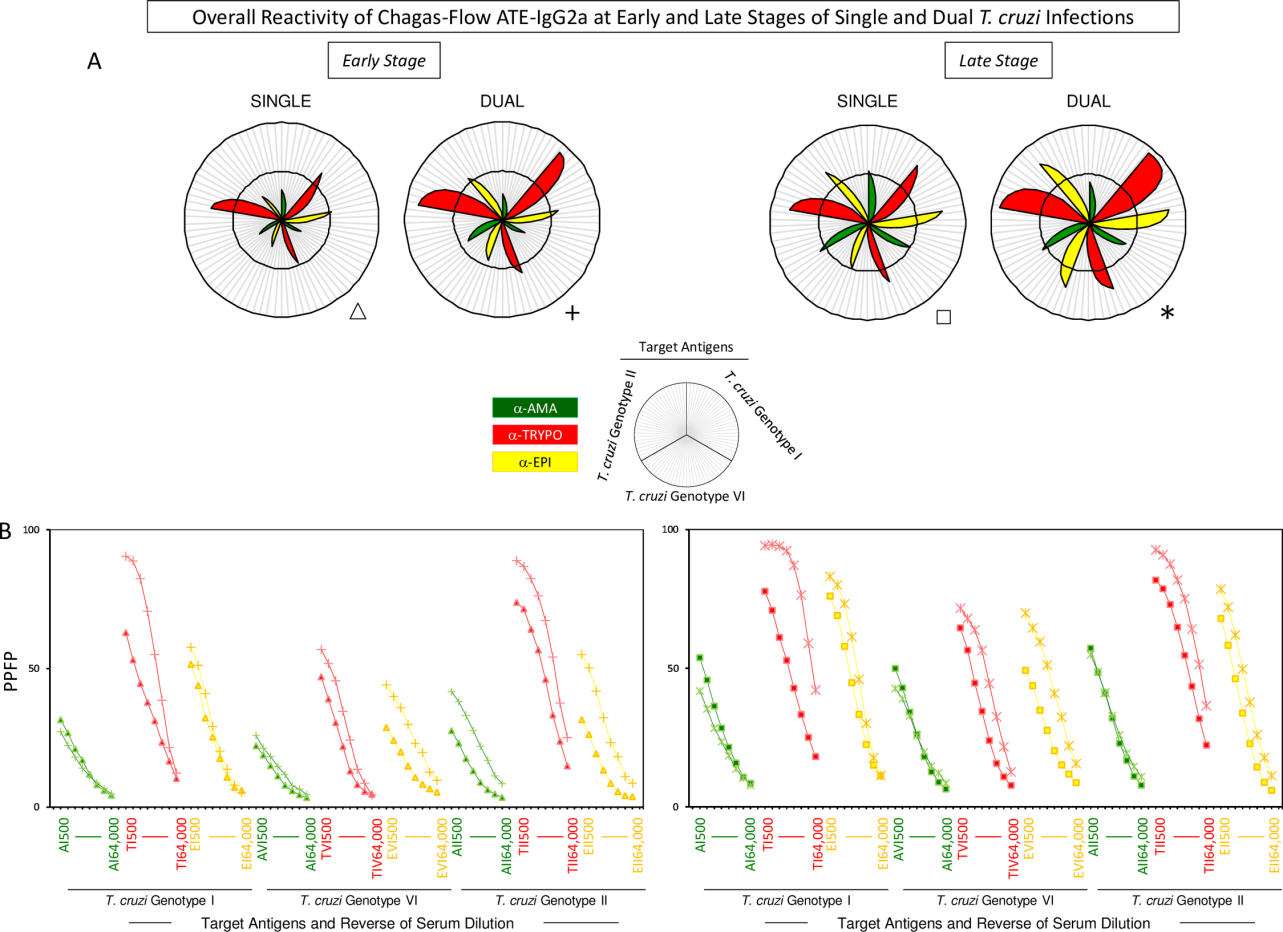
Overall Chagas-Flow ATE-IgG2a reactivity at early and late stages of single and dual *T*. *cruzi* infections. (A) Panoramic reactivity profile of sera samples at early stage of single (Triangle, n = 50) and dual (Cross, n = 60) as well as at late stage of single (Square, n = 93) and dual (Asterisk, n = 107) *T*. *cruzi* infections. The IgG2a reactivity was evaluated along the titration curve with each target-antigens amastigote (AMA = green), trypomastigote (TRYPO = red) and epimastigote (EPI = yellow) from TcI, TcVI and TcII genotypes of *T*. *cruzi*. The results are shown in radar charts and expressed as mean of percentage of positive fluorescent parasites (PPFP). (B) Titration curves at eight serum dilutions (1:500 to 1:64,000) using target-antigens AI, AVI and AII; TI, TVI and TII and EI, EVI and EII were assessed at early and late stages of single and dual *T*. *cruzi* infections.

**Fig 3:**
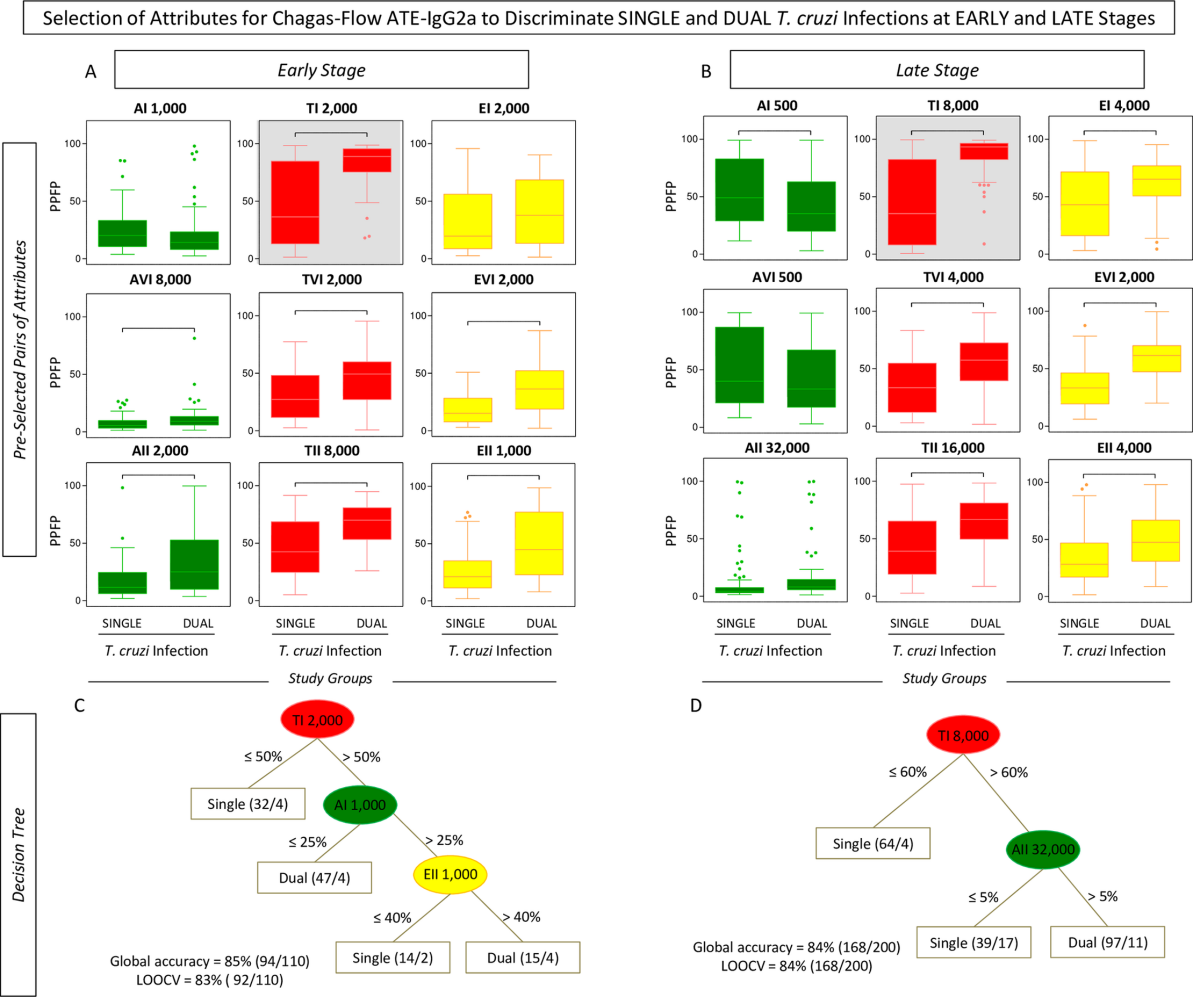
Selection of attributes for Chagas-Flow ATE-IgG2a to discriminate single and dual *T*. *cruzi* infections at early and late stages. (A) Comparative analysis of IgG2a reactivity at early stage of single (n = 50) and dual (n = 60) *T*. *cruzi* infections, using pre-selected pairs of attributes, including: AI 1,000; AVI 8,000; AII 2,000; TI 2,000; TVI 2,000; TII 8,000; EI 2,000; EVI 2,000 and EII 1,000. (B) Comparative analysis of IgG2a reactivity at late stage of single (n = 93) and dual (n = 107) *T*. *cruzi* infections, using pre-selected pairs of attributes, including: AI 500; AVI 500; AII 32,000; TI 8,000; TVI 4,000; TII 16,000; EI 4,000; EVI 2,000 and EII 4,000. The results are expressed as median of PPFP values in box plot format with the outliers showed by shaded dots. Comparative analyses were performed by the Kruskal-Wallis/Dunn’s post test. Significant differences were considered at p<0.05 and highlighted by connecting lines. The light gray background highlights the pairs of attributes (“target antigen and serum dilution”) with the higher performance to discriminate single and dual *T*. *cruzi* infections at early and late stages. Decision trees were constructed using the sets of attributes (“target-antigen and serum dilution/cut-off”) to create algorithms (root and branches) to classify individual samples from single and dual *T*. *cruzi* infections at (C) early and (D) late stages. Global accuracy and leave-one-out-cross-validation-LOOCV are provided in the Figure.

Similar approaches were employed to select the sets of attributes (“target-antigen and serum dilution/cut-off”) for differential diagnosis of single and dual at early and late *T*. *cruzi* infections (Figs 2 and 3).

The overall reactivity profile of dual infection demonstrated that regardless the stage, the mean reactivity to “T” was the highest. In general, reactivity to “A” and “E” were higher in late stage in both single and dual infections. Reactivity of late dual infection samples to “E” was even higher (Fig 2A and 2B).

Pairs of attributes (“target-antigen and serum dilution”) were selected to distinguish single and dual *T*. *cruzi* infections during early and late stages. In this sense, “TI 2,000”; “AVI 8,000”; “TVI 2,000”; “EVI 2,000”; “AII 2,000”; “TII 8,000” and “EII 1,000” were pre-selected to discriminate single from dual infections at early stage (Fig 3A). On the other hand, “AI 500”; “TI 8,000”; “EI 4,000”; “TVI 4,000”; “EVI 2,000”; “TII 16,000” and “EII 4,000” presented the highest ability to distinguish single and dual *T*. *cruzi* infections at late stage (Fig 3B).

Comparative analyses, based on the modular distance between the mean reactivity (PPFP) along the titration curves and on box plot charts of selected target antigens, indicated “TI 2,000” and “TI 8,000” as the top attributes to distinguish single and dual *T*. *cruzi* infections at early and late stages, respectively (Fig 3A and 3B—gray background).

The ROC curve analyses were used to calculated cut-off edges and define sets of attributes (“target-antigen and serum dilution/cut-off”) to construct decision tree algorithms (Fig 3C and 3D). The decision tree comprised of the following sets “TI 2,000/50%” (root), “AI 1,000/25%” (first branch) and “EII 1,000/40%” (second branch) segregated individual samples from single *vs* dual *T*. *cruzi* infections at early stage, with elevated global accuracy (85.0%, LOOCV = 84.0%) (Fig 3C). Moreover, the algorithm comprised of “TII 8,000/60%” (root) and “AII 32,000/5%” (branch) identified individual samples from single *vs* dual *T*. *cruzi* infections at late stage, also with elevated global accuracy (84.0%, LOOCV = 84.0%) (Fig 3D).

### Performance of Chagas-Flow ATE-IgG2a for genotype-specific diagnosis of *T*. *cruzi* infections

**Fig 4:**
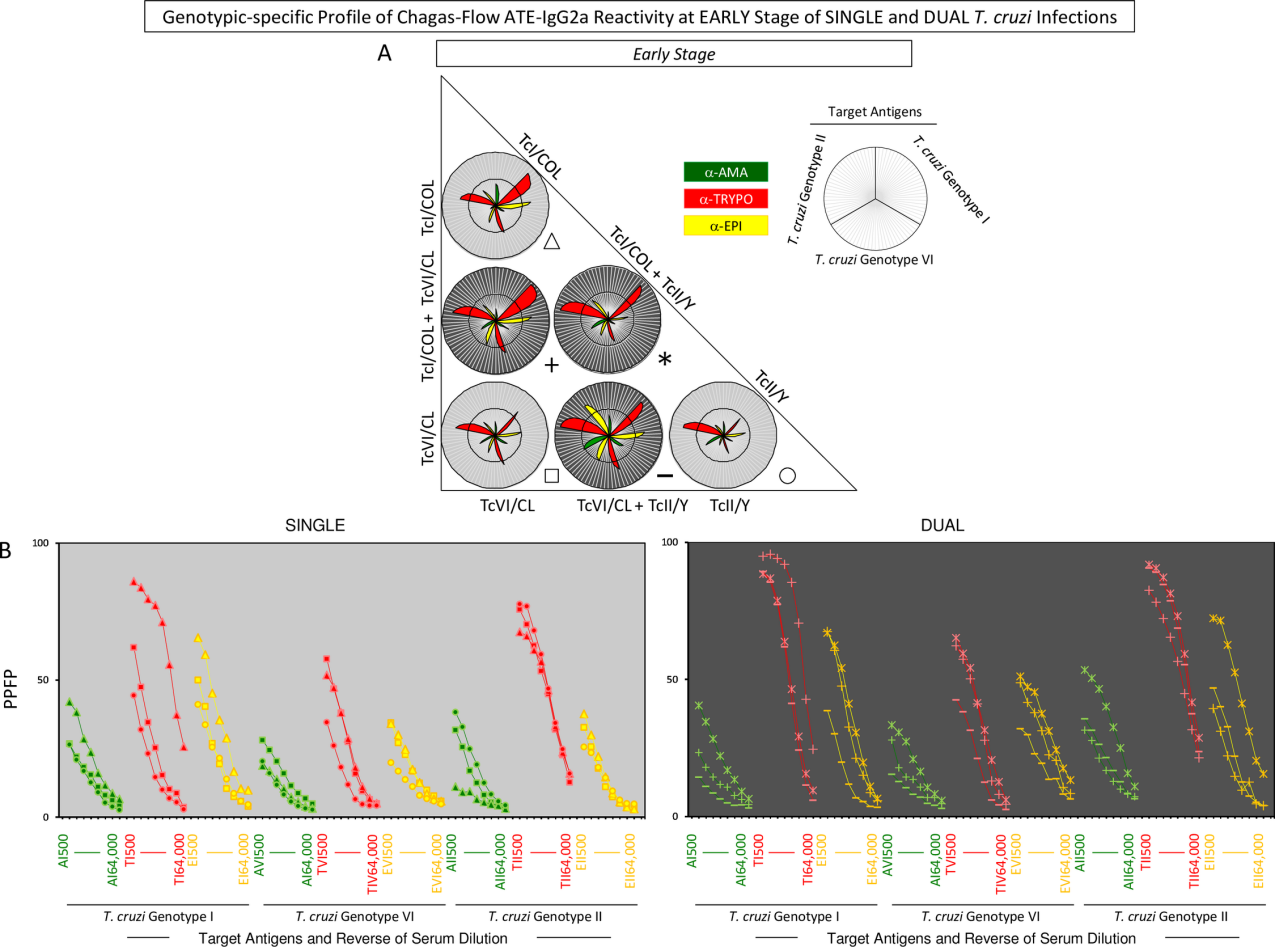
Genotype-specific profile of Chagas-Flow ATE-IgG2a reactivity at early stage of single and dual *T*. *cruzi* infections. (A) Overall reactivity profile of sera samples at early stage of single (TcI/COL, n = 16; TcVI/CL, n = 15 and TcII/Y, n = 19) and dual (TcI/COL+TcVI/CL, n = 16; TcVI/CL+TcII/Y, n = 24 and TcI/COL+TcII/Y, n = 20) *T*. *cruzi* infections with distinct genotypes. The IgG2a reactivity was evaluated along the titration curve with each target-antigens amastigote (AMA = green), trypomastigote (TRYPO = red) and epimastigote (EPI = yellow) from TcI, TcVI and TcII genotypes of *T*. *cruzi*. The results are shown on radar charts and expressed as mean of percentage of positive fluorescent parasites (PPFP). (B) Titration curves were assessed at eight serum dilutions (1:500 to 1:64,000) using target-antigens AI, AVI and AII; TI, TVI and TII along with EI, EVI and EI at early stage of single [TcI/COL (triangle), TcVI/CL (square), TcII/Y (circle)] and dual [TcI/COL+TcVI/CL (cross), TcVI/CL+TcII/Y (dash), TcI/COL+TcII/Y (asterisk)] *T*. *cruzi* infections.

**Fig 5:**
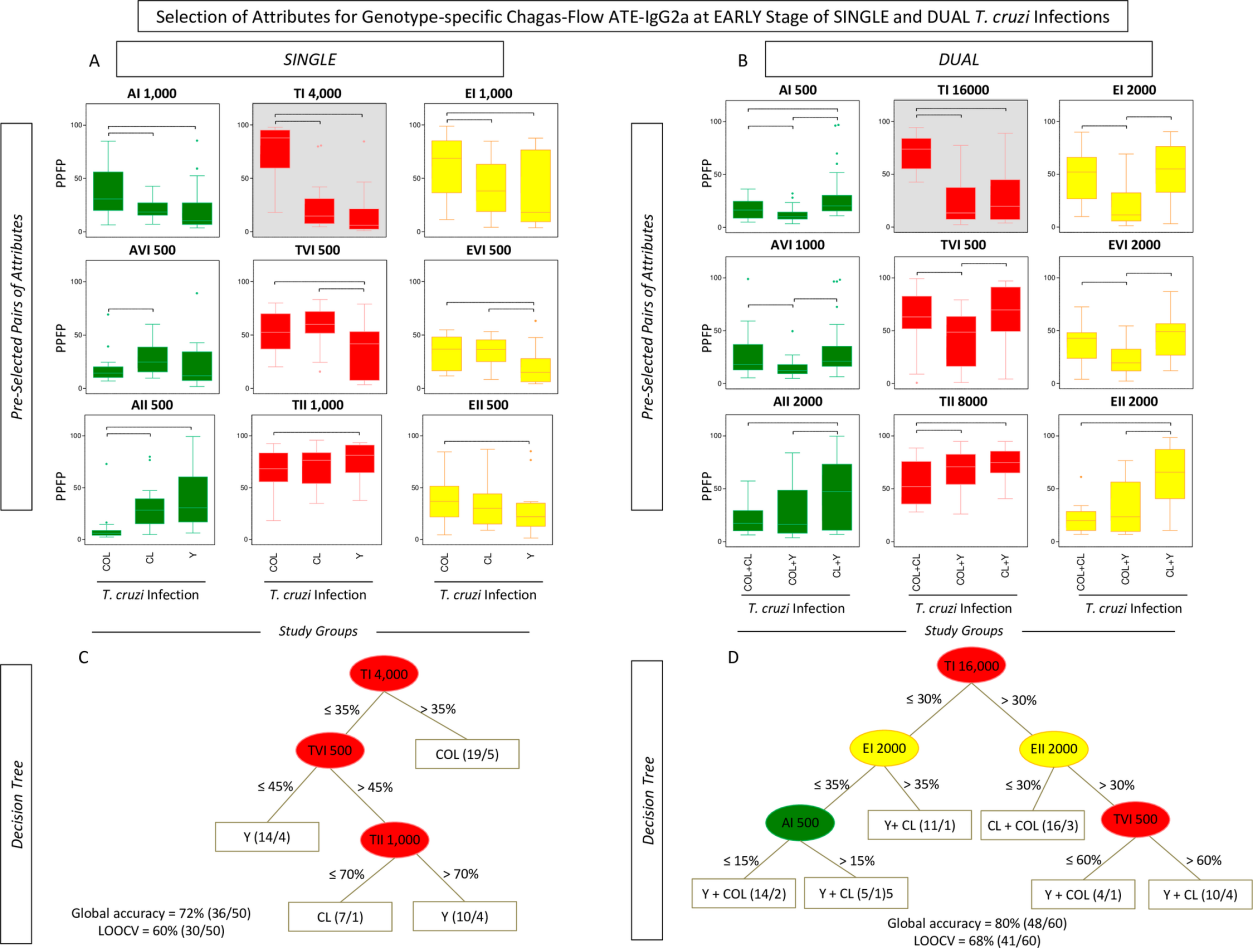
Selection of attributes for genotype-specific Chagas-Flow ATE-IgG2a at early stage of single and dual *T*. *cruzi* infections. (A) Comparative analysis of IgG2a reactivity at early stage of single infection with distinct *T*. *cruzi* genotypes (TcI/COL, n = 16; TcVI/CL, n = 15 and TcII/Y, n = 19) using pre-selected pairs of attributes, including: AI 1,000; AVI 500; AII 500; TI 4,000; TVI 500; TII 1,000; EI 1,000; EVI 500 and EII 500. (B) Comparative analysis of IgG2a reactivity at early stage of dual infection with distinct *T*. *cruzi* genotypes (TcI/COL+TcVI/CL, n = 16; TcVI/CL+TcII/Y, n = 24 and TcI/COL+TcII/Y, n = 20) using pre-selected pairs of attributes, including: AI 500; AVI 1,000; AII 2,000; TI 16,000; TVI 500; TII 8,000; EI 2,000; EVI 2,000 and EII 2,000. The results are expressed as median PPFP values in box plot format with the outliers showed by shaded dots. Comparative analyses were performed by the Kruskal-Wallis/Dunn’s post test. Significant differences were considered at p<0.05 and highlighted by connecting lines. The light gray background highlights the pairs of attributes (“target antigen and serum dilution”) with the higher performance for the genotype-specific diagnosis at early stage of single and dual *T*. *cruzi* infections. Decision trees were constructed using the sets of attributes (“target-antigen and serum dilution/cut-off”) to create algorithms (root and branches) to classify at early stage, individual samples from (C) single and (D) dual *T*. *cruzi* infections. Global accuracy and leave-one-out-cross-validation-LOOCV are provided in the Figure.

**Fig 6:**
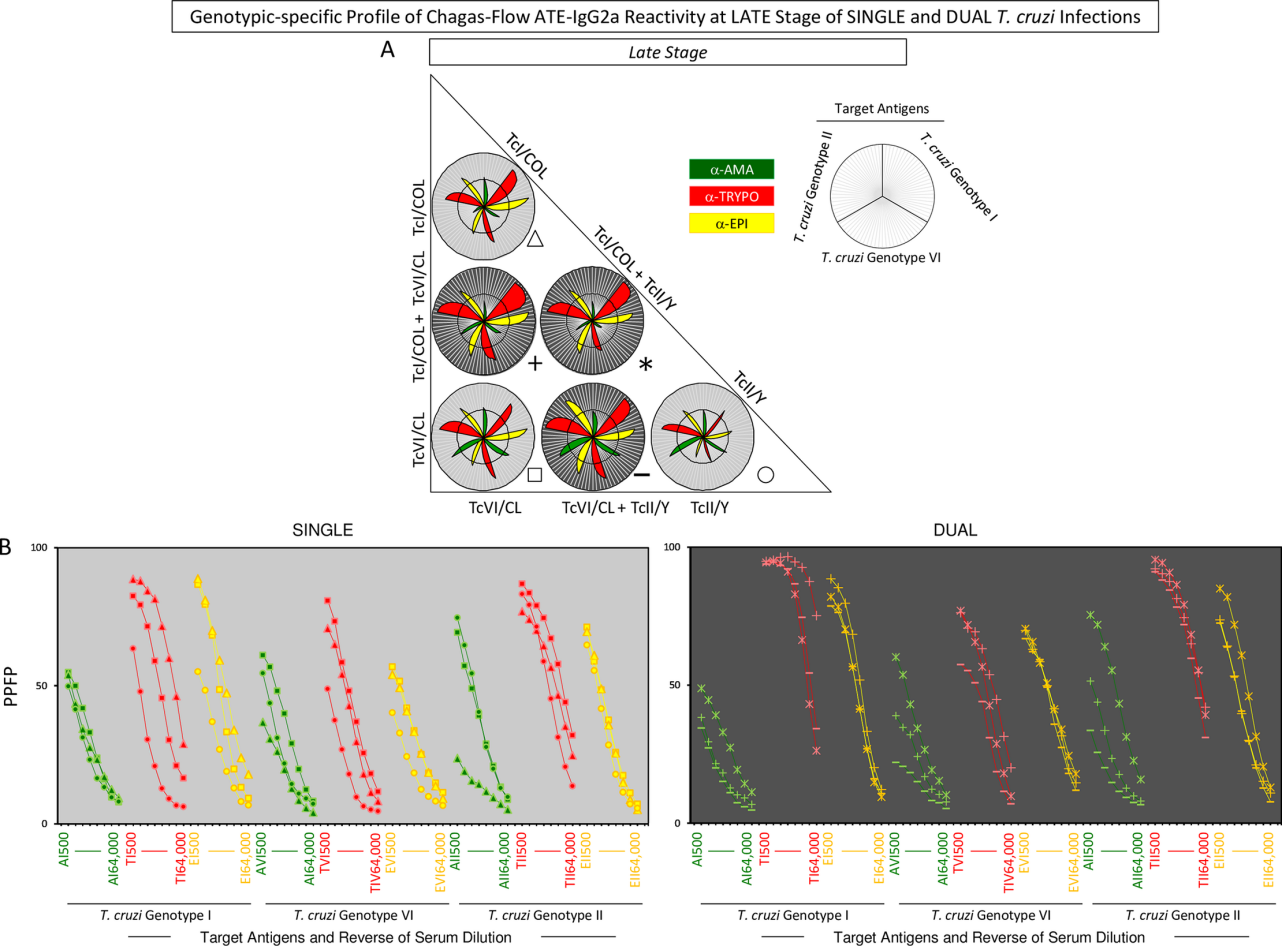
Genotype-specific profile of Chagas-Flow ATE-IgG2a reactivity at late stage of single and dual *T*. *cruzi* infections. (A) Overall reactivity profile of sera samples at late stage of single (TcI/COL, n = 29; TcVI/CL, n = 29 and TcII/Y, n = 35) and dual (TcI/COL+TcVI/CL, n = 28; TcVI/CL+TcII/Y, n = 43 and TcI/COL+TcII/Y, n = 36) *T*. *cruzi* infections with distinct genotypes. The IgG2a reactivity was evaluated along the titration curve with each target-antigens amastigote (AMA = green), trypomastigote (TRYPO = red) and epimastigote (EPI = yellow) from TcI, TcVI and TcII genotypes of *T*. *cruzi*. The results are shown on radar charts and expressed as mean of percentage of positive fluorescent parasites (PPFP). (B) Titration curves were assessed at eight serum dilutions (1:500 to 1:64,000) using target-antigens AI, AVI and AII; TI, TVI and TII along with EI, EVI and EI at late stage of single [TcI/COL (triangle), TcVI/CL (square), TcII/Y (circle)] and dual [TcI/COL+TcVI/CL (cross), TcVI/CL+TcII/Y (dash), TcI/COL+TcII/Y (asterisk)] *T*. *cruzi* infections.

**Fig 7:**
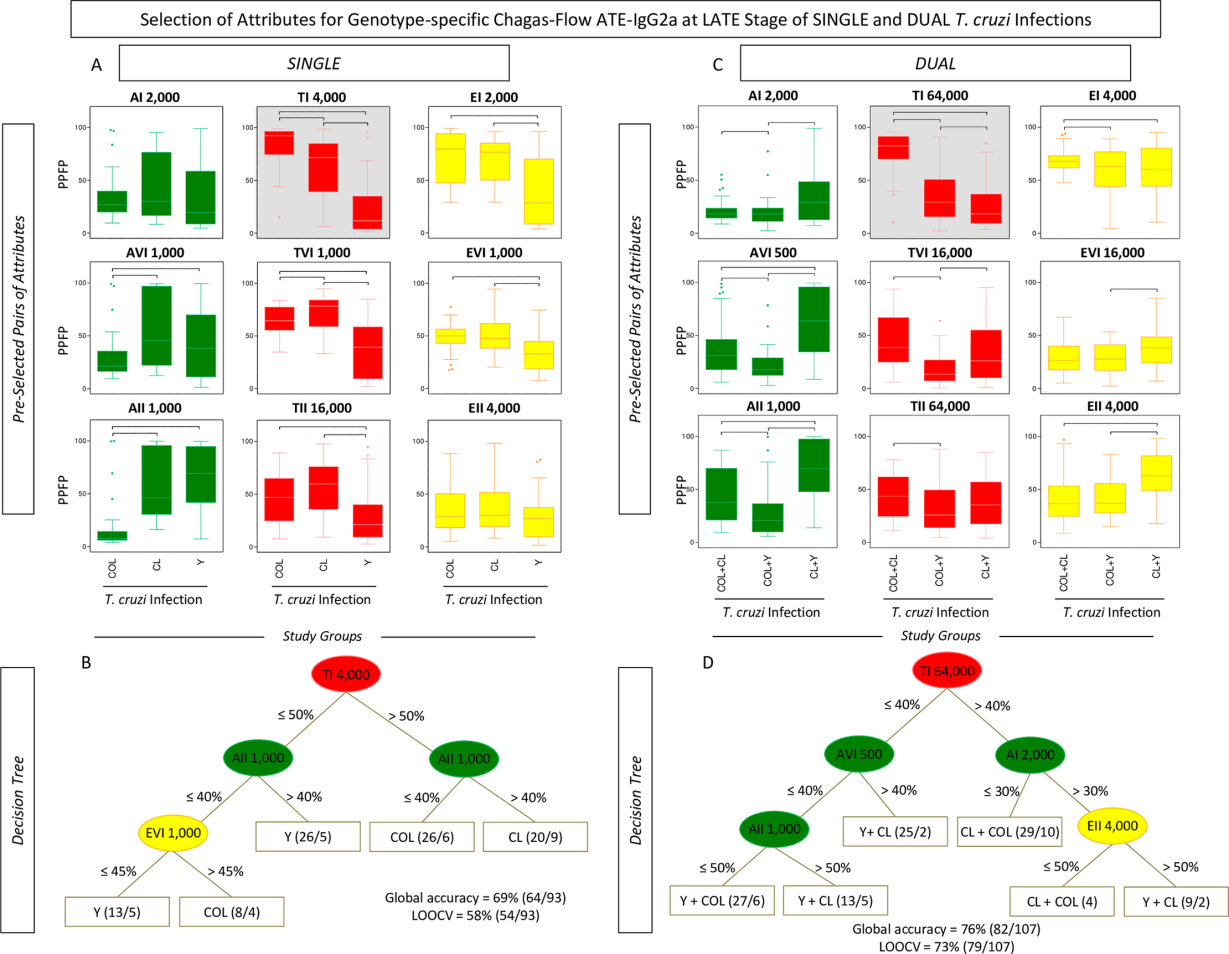
Selection of attributes for genotype-specific Chagas-Flow ATE-IgG2a at late stage of single and dual *T*. *cruzi* infections. (A) Comparative analysis of IgG2a reactivity at late stage of single infection with distinct *T*. *cruzi* genotypes (TcI/COL, n = 29; TcVI/CL, n = 29 and TcII/Y, n = 35) using pre-selected pairs of attributes, including: AI 2,000; AVI 1,000; AII 1,000; TI 4,000; TVI 1,000; TII 16,000; EI 2,000; EVI 1,000 and EII 4,000. (B) Comparative analysis of IgG2a reactivity at late stage of dual infection with distinct *T*. *cruzi* genotypes (TcI/COL+TcVI/CL, n = 28; TcVI/CL+TcII/Y, n = 43 and TcI/COL+TcII/Y, n = 36) using pre-selected pairs of attributes, including: AI 2,000; AVI 500; AII 1,000; TI 64,000; TVI 16,000; TII 64,000; EI 4,000; EVI 16,000 and EII 4,000. The results are expressed as median PPFP values in box plot format with the outliers showed by shaded dots. Comparative analyses were performed by the Kruskal-Wallis/Dunn’s post test. Significant differences were considered at p<0.05 and highlighted by connecting lines. The light gray background highlights the pairs of attributes (“target antigen and serum dilution”) with the higher performance for the genotype-specific diagnosis at late stage of single and dual *T*. *cruzi* infections. Decision trees were constructed using the sets of attributes (“target-antigen and serum dilution/cut-off”) to create algorithms (root and branches) to classify at late stage, individual samples from (C) single and (D) dual *T*. *cruzi* infections. Global accuracy and leave-one-out-cross-validation-LOOCV are provided in the Figure.

Aiming at providing a feasible approach to employ the overall Chagas-Flow ATE-IgG2a methodology to genotype-specific diagnosis, the overall reactivity observed at early and late stages of single and dual infections with distinct *T*. *cruzi* genotypes was first evaluated by radar charts followed by the construction of decision trees (Figs 4, 5, 6 and 7).

A panoramic snapshot provided by the reactivity of sera samples during early and late stages of single and dual *T*. *cruzi* infections (Figs 4 and 6). The results demonstrated that the reactivity to “TII” was high regardless the stage (early or late) and type of infection (single or dual), re-enforcing the use of this attribute for universal diagnosis purposes. Additionally, higher reactivity to “TI” was usually associated with single infection with TcI/COL or dual infection. Conversely, the reactivity to “TI” was lower in TcII/Y infection. Moreover, specifically at late stage, the reactivity to “A” was lower in single or dual infections involving TcI/COL (Figs 4 and 6).

Line plots of mean reactivity profiles and box plot charts compiling the median PPFP values subsidize the pre-selection of pairs of attributes (“target-antigen and serum dilution”) with better performance for genotype-specific diagnosis at early stage of single and dual *T*. *cruzi* infections. The pair of attributes “AI 1,000”; “TI 4,000”; “EI 1,000”; “AVI 500”; “TVI 500”; “EVI 500”; “AII 500”; “TII 1,000” and “EII 500” were pre-selected based on their applicability of genotype-specific diagnosis at early stage of single *T*. *cruzi* infection (Fig 5A). The attributes “AI 500”; “TI 16,000”; “EI 2,000”; “AVI 1,000”; “TVI 500”; “EVI 2,000”; “AII 2,000”; “TII 8,000” and “EII 2,000” were pre-selected for genotype-specific diagnosis at early stage of dual *T*. *cruzi* infection (Fig 5B). The pairs of attributes with the most promising performance for genotypic-specific diagnosis at early stage of single and dual *T*. *cruzi* infections were “TI 4,000” and “TI 16,000”, respectively (Fig 5A and 5B).

The global mean or median values were used as specific cut-off edges to define sets of attributes (“target-antigen and serum dilution/cut-off”) to construct decision tree algorithms (Fig 5C and 5D). The sets “TI 4,000/35%” (root), “TVI 500/45%” (first branch) and “TII 1,000/70%” (second branch) were selected to compose the decision tree that classifies individual samples at early stage of single infection with a moderate global accuracy (72.0%, LOOCV = 60.0%) (Fig 5C). The algorithm composed by “TI 16,000/30%” (root), “EI 2,000/35%” and “EII 2,000/35%” (first branches) along with “AI 500/15%” and “TVI 500/60%” (second branches) classified individual samples at early stage of dual *T*. *cruzi* infection, also with a moderate global accuracy (80.0%, LOOCV = 68.0%) (Fig 5D).

Titration curves of mean reactivity profile and box plot charts displaying median PPFP values were used to pre-select the pairs of attributes (“target-antigen and serum dilution”) applicable to the genotype-specific diagnosis at late stage of single and dual *T*. *cruzi* infections (Fig 7A). The attributes “TI 4,000”; “EI 2,000”; “AVI 1,000”; “TVI 1,000”; “EVI 1,000”; “AII 1,000” and “TII 16,000” presented better performance for genotype-specific diagnosis at late stage of single *T*. *cruzi* infection (Fig 7A). Moreover, “AI 2,000”; “TI 64,000”; “EI 4,000”; “AVI 500”; “TVI 16,000”; “EVI 16,000”; “AII 1,000”; “TII 64,000” and “EII 4,000” were pre-selected for genotype-specific diagnosis at late stage of dual *T*. *cruzi* infection (Fig 7B). The “TI 4,000” and “TI 64,000” were elected as the “top” pairs of attributes for genotype-specific diagnosis at late stage of single and dual *T*. *cruzi* infections, respectively (Fig 7A and 7B).

The global mean or median values were used as specific cut-off edges to define sets of attributes (“target-antigen and serum dilution/cut-off”) to build decision trees for genotype-specific diagnosis at late stage of single and dual *T*. *cruzi* infections (Fig 7C and 7D). The “TI 4,000/50%” (root), “AII 1,000/40%” (first branches) and “EVI 1,000/45%” (second branch) to classify individual samples at late stage of single *T*. *cruzi* infection, with moderate global accuracy (69.0%, LOOCV = 58.0%) (Fig 7C). Besides, the algorithm proposed for genotype-specific diagnosis at late stage of dual infection include the attributes “TI 64,000/40%” (root), “AVI 500/40%” and “AI 2,000/30%” (first branches) along with “AII 1,000/50%” and “EII 4,000/50%” (second branches) to categorize the individual samples also with a moderate global accuracy (76.0%, LOOCV = 73.0%) (Fig 7D).

### Overall performance of Chagas-Flow ATE-IgG2a for differential diagnosis of *T*. *cruzi* infection

**Fig 8:**
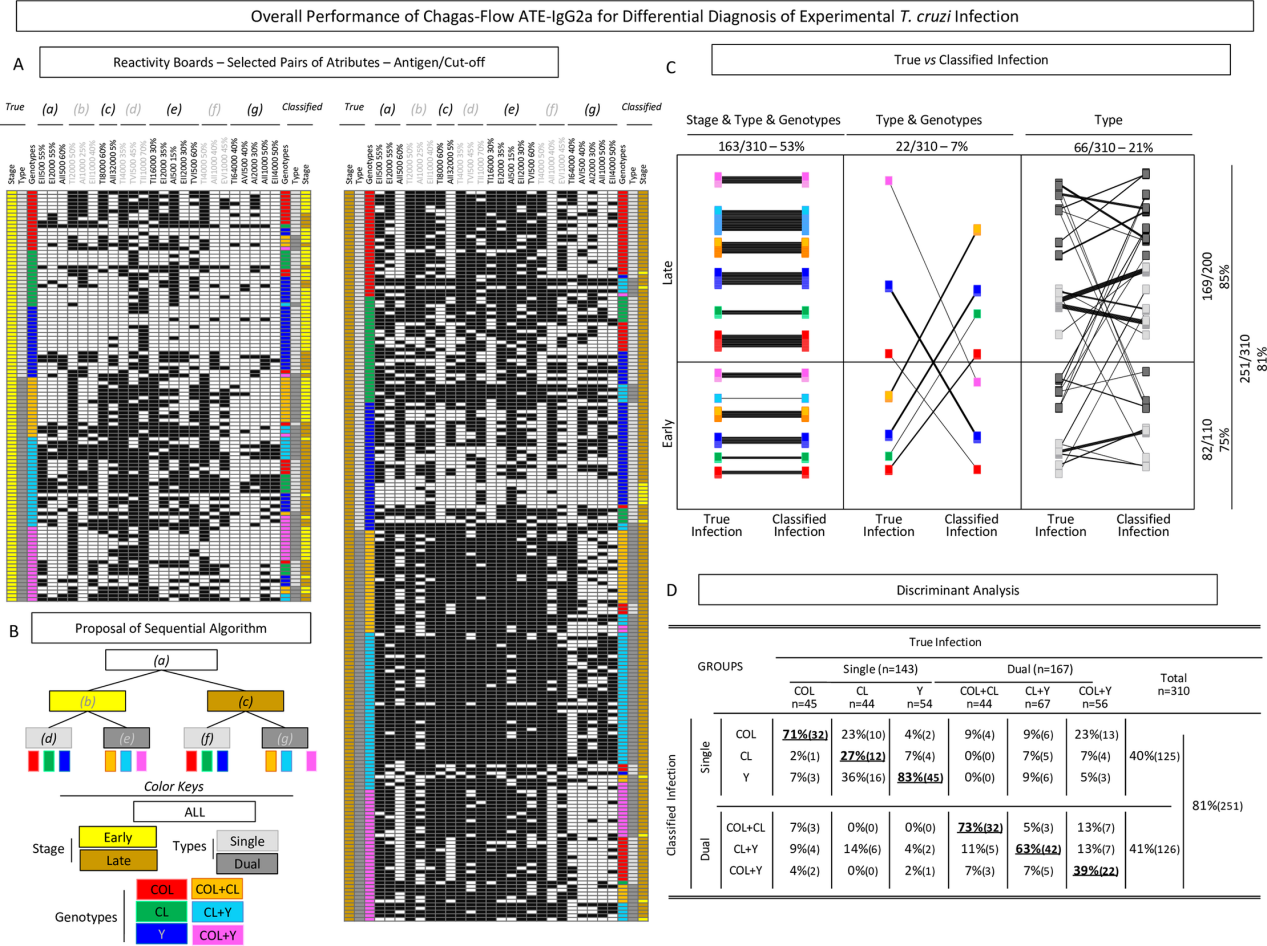
Overall performance of Chagas-Flow ATE-IgG2a for differential diagnosis of experimental *T*. *cruzi* infection. (A) Reactivity boards were employed to display the overall profile of Chagas-Flow ATE-IgG2a applied to the differential diagnosis of *T*. *cruzi* infection at early (left diagram) and late (right diagram), using the selected sets of attributes (“target-antigen and serum dilution/cut-off”) determined by the decision trees. (B) Sequential algorithms were applied to yield the “classified infection” status to each individual sample, referred as: “a” for early *vs* late; “b” for single *vs* dual infections at early stage; “c” for single *vs* dual infections at late stage; “d” for genotype-specific diagnosis at early stage of single infection; “e” for genotype-specific diagnosis at early stage of dual infection; “f” for genotype-specific diagnosis at late stage of single infection and “g” for genotype-specific diagnosis at late stage of dual infection. A color key was provided to identify early (yellow), late (brown), single (light gray), dual (dark gray), TcI/COL (red), TcVI/CL (green), TcII/Y (dark blue), TcI/COL+TcVI/CL (orange), TcVI/CL+TcII/Y (light blue) and TcI/COL+TcII/Y (pink). (C) Scatter graphs showed correspondence between each pair of “true” *vs* “classified” infection indicated by connecting lines and the percentage of agreement for “Stage & Type & Genotype”; “Type & Genotype” and “Type” of infection. (D) Discriminant analyses of combined Chagas-Flow ATE-IgG2a for genotype-specific diagnosis including *T*. *cruzi* single infection (COL, CL and Y) and dual infection (COL+CL, CL+Y and COL+Y). The global accuracy is provided in the Figure.

The overall performance of Chagas-Flow ATE-IgG2a was estimated after gathering the reactivity profile of individual samples using the selected sets of attributes (Fig 8A).

Following, the sequential algorithms (referred as “a”, “b”, “c”, “d”, “e”, “f” and “g”) were applied to yield the “classified infection” status to each individual sample (Fig 8B). Subsequently the results of each pair of “true infection” *vs* “classified infection” were compared to estimate the percentage of agreement (Fig 8C).

Data analysis showed that the “Stage & Type & Genotypes” were classified correctly in 163 samples (53%). Moreover, the “Type & Genotypes”, were classified correctly in 22 samples (7%), whereas the “Type” were classified correctly in 66 samples (21%) (Fig 8C). Together the results demonstrated that 251 out of 310 samples (81%) were classified correctly (Fig 8C). Our data demonstrated that 23 out of 310 samples were extreme outliers (7%), misclassified regarding to the stage, type and genotype, most from early infection (n = 22). The remaining 36 samples were misclassified according to the type and genotype, most of them form late infection (n = 30).

Detailed discriminant analysis of Chagas-Flow ATE-IgG2a applied to the genotype-specific diagnosis of *T*. *cruzi* infection, regardless the stage of infection is provided in the Fig 8D. In general, 71% of “true infection” with TcI/COL and 83% of “true infection” with TcII/Y were classified correctly, whereas only 27% of “true infection” with TcVI/CL received a correct classification status of single infection (Fig 8D). On the other hand, 73% of “true infection” with TcI/COL+TcVI/CL and 63% of “true infection” with TcVI/CL+TcII/Y were classified correctly, whereas only 39% of “true infection” with TcI/COL+TcII/Y received a correct classification status of dual infection (Fig 8D). Together, the overall performance of Chagas-Flow ATE-IgG2a for differential diagnosis of *T*. *cruzi* infection reached an elevated global accuracy (81%) (Fig 8D).

Discriminant analysis at early and late stages of infection is provided in the S2 Fig. In general, the performance of Chagas-Flow ATE-IgG2a applied to the genotype-specific diagnosis of *T*. *cruzi* infection was higher at late stage (85%) as compared to early (75%) stage of infection (S2 Fig). Generally, at early stage, the TcII/Y (90%) infection was better classified, whereas at late stage, the TcI/COL (80%), TcII/Y (80%) and TcVI/CL+TcII/Y (83%) infections received the higher percentage of agreement between “true *vs* classified” status (S2 Fig).

## Discussion

Recently, an innovative technique was developed with applicability for universal and genotype-specific diagnosis of single experimental *T*. *cruzi* infection, in the chronic phase, named Chagas-Flow ATE-IgG2a [32], and originally proposed by Alessio *et al*. (2014) [31]. Chagas-Flow ATE-IgG2a is perform with distinct *T*. *cruzi* genotypes as target antigens, employing parasites strains from the three major genotypes (TcI, TcVI and TcII) associated with human Chagas disease in Latin America [21, 36, 37]. Our previous results demonstrated an excellent performance of Chagas-Flow ATE-IgG2a assay for universal diagnosis of experimental *T*. *cruzi* infection, with 100% sensitivity and specificity. Further, the method showed a good performance for genotype-specific diagnosis of single experimental *T*. *cruzi* infection during chronic phase, with accuracy of 94% to segregate TcI and TcII infections [32]. Thus, the present work aims to extend the early studies carried out by Alessio *et al*. (2017) [32], in order to improve the Chagas-Flow ATE-IgG2a for genotype-specific diagnosis of single and dual experimental *T*. *cruzi* infections, in early and late stages. The opportunity to investigate the antibody response at distinct stages of experimental *T*. *cruzi* infections intended to typify, in practical terms, the status of Chagas disease observed in children or young adults (early) as well as in middle age adults (late).

Endemic areas of Chagas disease allow individuals to undergo multiple re-infections that can result in different *T*. *cruzi* populations throughout the course of the infection [17, 38–42]. At mixed infections, distinct genotypes of *T*. *cruzi* may be found in blood and tissues, according to the phase or time of infection [13, 17, 19]. Indeed, Sales-Campos *et al*. (2014) [19] have demonstrated that during TcI/TcII mixed infections, TcII strains predominates in blood samples during both acute and chronic phases. On the other hand, in tissues samples, TcII strains were more prevalent during the acute phase, while TcI strains were more commonly identified during chronic phase of infection. Conversely, other studies have shown that TcI predominate over TcII strains in blood and tissue samples during acute and chronic infections [43]. In fact, the survival of one specific parasite population in blood can be associated with its capacity to escape from the host immune response [44, 45]. Thus, the standardization of the Chagas-Flow ATE-IgG2a methodology for the genotype-specific diagnosis at early and late stages for single and dual *T*. *cruzi* infection is extremely important to elucidate the genetic groups of *T*. *cruzi* which may result in distinct disease outcome.

Chagas-Flow ATE-IgG2a showed a good performance to discriminate early and late *T*. *cruzi* infections, with an elevated global accuracy (81%) (Fig 1). The correct knowledge regarding the stage of *T*. *cruzi* infection is essential since previous studies already demonstrated that distinct *T*. *cruzi* genotypes in host present particular behavior according to the time of infection [46–48]. Moreover, Toledo *et al*. (2003) [47] verified differences among stocks of *T*. *cruzi* in the response to treatment according to the genotype of the parasite and phase of infection. Stocks of genotype 39 (hybrids) were resistant to itraconazole treatment in acute phase and partially resistant in the chronic phase. On the other hand, stocks of genotype 32 (TcII) were partially susceptible to itraconazole treatment in acute phase and susceptible in the chronic phase. Therefore, Chagas-Flow ATE-IgG2a arises as a new tool that can distinguish early and late *T*. *cruzi* infections.

Our data demonstrated high reactivity of serum samples with the “T” antigen was high in early and late stages of *T*. *cruzi* infection. Conversely, low reactivity with the “A” antigen was observed during early stage which enhances in late infection (Fig 1). These results are consistent with the biological cycle of *T*. *cruzi* [49, 50]. Moreover, serum samples from *T*. *cruzi* infected mice showed high reactivity both in early and late stages with “TII” target antigen, demonstrating again to be a good antigen for universal diagnosis of *T*. *cruzi* infection, as observed previously by our group [32]. Additionally, high reactivity of the serum samples from mice in late stage compared to early stage was observed (Fig 1), suggesting an association of a better specificity with the establishment of the host immune response and therefore with the greater reactivity of antibodies during late infection [51–54].

Chagas-Flow ATE-IgG2a demonstrated an essential ability to discriminate single and dual *T*. *cruzi* infections with an elevated global accuracy of 85% in early stage and 84% in late stage (Figs 2 and 3). The capacity to differentiate between single and dual infections can provide better approaches for the appropriate treatment [14, 15]. A remarkable finding of the present work was the higher reactivity of sera samples from dual infection as compared to single infection, in both early and late stages (Figs 2 and 3). These findings reflect the fact that during dual infections the host immune system is exposed to a broader antigenic repertoire that leads to higher antibody response [51–54].

This study is pioneer in the development of a serological method by flow cytometry able to distinguish single and dual *T*. *cruzi* infections during both early and late stages. Serological methods previous standardized for genotype-specific diagnosis evaluated particularly the reactivity of serum samples from single *T*. *cruzi* infection and not considering the phase of infection [27–30]. In general, the available serological approaches are not suitable to detect mixed infections in human samples from different countries [29]. One potential application of Chagas-Flow ATE is the putative genotypic characterization of Chagas disease in distinct geographic areas. Serodiscordant results are frequently observed when conventional serological methods are employed in endemic areas where TcI genotype is predominant [55]. Moreover, the existence of universal specific antigen in the Chagas-Flow ATE (TII antigen) could be very important to guarantee the sensitivity of Chagas disease diagnosis in areas with high variability of *T*. *cruzi* genotypes due to high number of immigrants from distinct endemic areas.

Chagas-Flow ATE-IgG2a showed a good performance for genotype-specific diagnosis of single and dual *T*. *cruzi* infections, both in early and late stages. During early stage, this methodology was able to differentiate single infection with a global accuracy of 72% and dual infection presenting a global accuracy of 80% (Fig 5). Regarding the late stage, this methodology was able to discriminate dual infection with a moderate global accuracy (76%) (Fig 7).

An interesting result highlights that in several algorithms of the decision trees, the root attribute was the “TI” target antigen (Figs 3, 5 and 7). The high reactivity of serum samples with “TI” can be associated with the predominant morphology of the parasite genotype (broad strains), and therefore with the better resistance to complement-mediated lyses, being a more immunogenic strain [56]. Also, single infection presented a similar profile both in early and late stages with mice serum samples infected with TcI/COL showing high reactivity with “TI” antigen and low reactivity with “AII’ antigen and mice serum samples infected with TcII/Y presenting high reactivity with “AII” antigen and low reactivity with “TI” and “EVI” antigens (Figs 5 and 7).

Some techniques of molecular biology are capable to detect mixed *T*. *cruzi* infections, such as: the ones used in the protocol of D´Ávilla *et al*. (2009) [22], which combines the analyses of the 24sα-LSU rDNA gene´s polymorphism [22, 57, 58], the polymorphism of mini-exon SL-IL intergenic spacer [59] as well as the profile of bands obtained after PCR-RFLP of the subunit II cytochrome oxidase gene [60]. Previous studies identified the mixed *T*. *cruzi* infections by kDNA PCR amplification and subsequent hybridization using DTU-specific probes (DTU-blot) [11, 40, 61, 62], using PCR-Real Time with Taq-Man probes (MTq-PCR) [63] and by the amplification of other *T*. *cruzi* genome targets, such as the cSC5D gene by PCR-RFL [64]. However, even using high complexity methodologies some of these approaches were not able to detect all the mixed infections present in hosts, as observed by the Chagas-Flow ATE-IgG2a.

The Chagas-Flow ATE-IgG2a identifies single and dual *T*. *cruzi* infections avoiding the need to isolate the parasite from the blood by low sensitivity techniques, such as hemoculture and xenodiagnosis, and *in vitro* cultures. Besides, molecular methods may lead to errors in the identification of genotypes that infect hosts, by selecting the populations. Indeed, previous studies demonstrated that parasite populations present in the blood hosts were distinct from those isolated by xenodiagnosis and cultured in culture media [11, 40, 62]. In addition, ORTIZ *et al*. (2015) [62] showed by kDNA PCR amplification and subsequent hybridization with DTU-specific probes, mixtures of TcI, TcII, TcV and TcVI DTUs in chronic chagasic host blood samples, although xenodiagnosis followed by the axenic cultures of the parasites identify mostly TcV.

The Chagas-Flow ATE-IgG2a seems to be an innovative serological method by flow cytometry with great potential for the genotype-specific diagnosis of single and dual *T*. *cruzi* infections. The analysis of the sequential algorithms of the decision trees demonstrated that this technique presented an accuracy of 81% as a complementary diagnostic test for the *T*. *cruzi* infection. Several advantages of Chagas-Flow ATE have been presented. The possible limitation of this method would be the fact that flow cytometers are not available in most laboratories in endemic areas. This putative limitation of using Chagas-Flow ATE in clinical studies can be overcome by the use of currently available portable flow cytometers or by establishing this method on reference laboratories. Moreover, as the Chagas-Flow ATE requires the use of live parasites (A and T antigens), the original proposal is to provide this innovative method at reference laboratory facilities. A perspective to overcome the use of live parasites, the second generation of the Chagas-Flow ATE, using purified or recombinant membrane antigens from distinct *T*. *cruzi* genotypes in a multiplex assay are under development. Additionally, it is important to mention that data generated from mouse model needs to be validate for human infections.

In conclusion, the Chagas-Flow ATE-IgG2a is a feasible tool to truly classify the serum samples infected with distinct *T*. *cruzi* genotypes, suggesting its applicability in the genotype-specific diagnosis of single and dual experimental *T*. *cruzi* infections. This methodology includes advantages such as high sensitivity and specificity, ability to differentiate serum samples during early and late stages, in single and dual infections and identify the specific genotype of *T*. *cruzi*. Moreover, the Chagas-Flow ATE can also be useful to establish clinical prognosis status of ongoing infection, since distinct *T*. *cruzi* genotypes has been associated with distinct severity of Chagas disease [21, 36, 42, 65]. An additional advantage of using the genotypic-specific Chagas-Flow ATE is its potential applicability for post-therapeutic monitoring, considering the distinct susceptibility of *T*. *cruzi* genotypes to etiological treatment [15, 47, 66]. In this context, the method would provide further information about post-therapeutic changes in the predominant *T*. *cruzi* DTU as compared to pre-treatment screening. The standardization of the Chagas-Flow ATE methodology for the genotype-specific diagnosis of human infection will be extremely important for further clinical purposes, epidemiological studies and post-therapeutic monitoring applications.

## Supporting information

S1 Fig Experimental design and methodological approaches. (A) Schematic flow chart overview of the experimental procedure Chagas-Flow ATE-IgG2a. Representative gating strategies to select the target antigens (amastigote-AMA = green, trypomastigote-TRYPO = red and epimastigote-EPI = yellow) and the histograms employed to quantify the percentage of positive fluorescent parasites (PPFP). (B) The compendium of the study population comprised of Swiss mice, categorized into subgroups referred as: early (yellow), late (brown), single (light gray), dual (dark gray), TcI/COL (red), TcVI/CL (green), TcII/Y (dark blue), TcI/COL+TcVI/CL (orange), TcVI/CL+TcII/Y (light blue) and TcI/COL+TcII/Y (pink). (C) Data mining approaches was achieved through the steps: Step 1 (algorithm for early & late stages); Step 2 (algorithms for single & dual infections); Step 3 (algorithm for genotypic-specific single & dual—early & late stages); Step 4 (assemblage sequential algorithm platform).
(TIF)

S2 Fig Discriminant analysis of combined Chagas-Flow ATE-IgG2a for genotype-specific diagnosis of *T. cruzi* infection. Discriminant analyses of combined Chagas-Flow ATE-IgG2a for genotype-specific diagnosis at (A) early and (B) late stages of *T*. *cruzi* single infection (COL, CL and Y) and dual infection (COL+CL, CL+Y and COL+Y). The global accuracy is provided in the Figure.
(TIF)

S3 Fig STARD flow diagram for studies reporting diagnostic accuracy. (TIF)

S1 Table STARD checklist for studies reporting diagnostic accuracy. (DOCX)
