# Supplementary figures and images for: Accomplishing the genotype-specific serodiagnosis of single and dual Trypanosoma cruzi infections by flow cytometry Chagas-Flow ATE-IgG2a
Source: PLoS Negl Trop Dis. 2018 Feb 20;12(2):e0006140. doi: 10.1371/journal.pntd.0006140 (PMC5843347; doi:10.1371/journal.pntd.0006140)

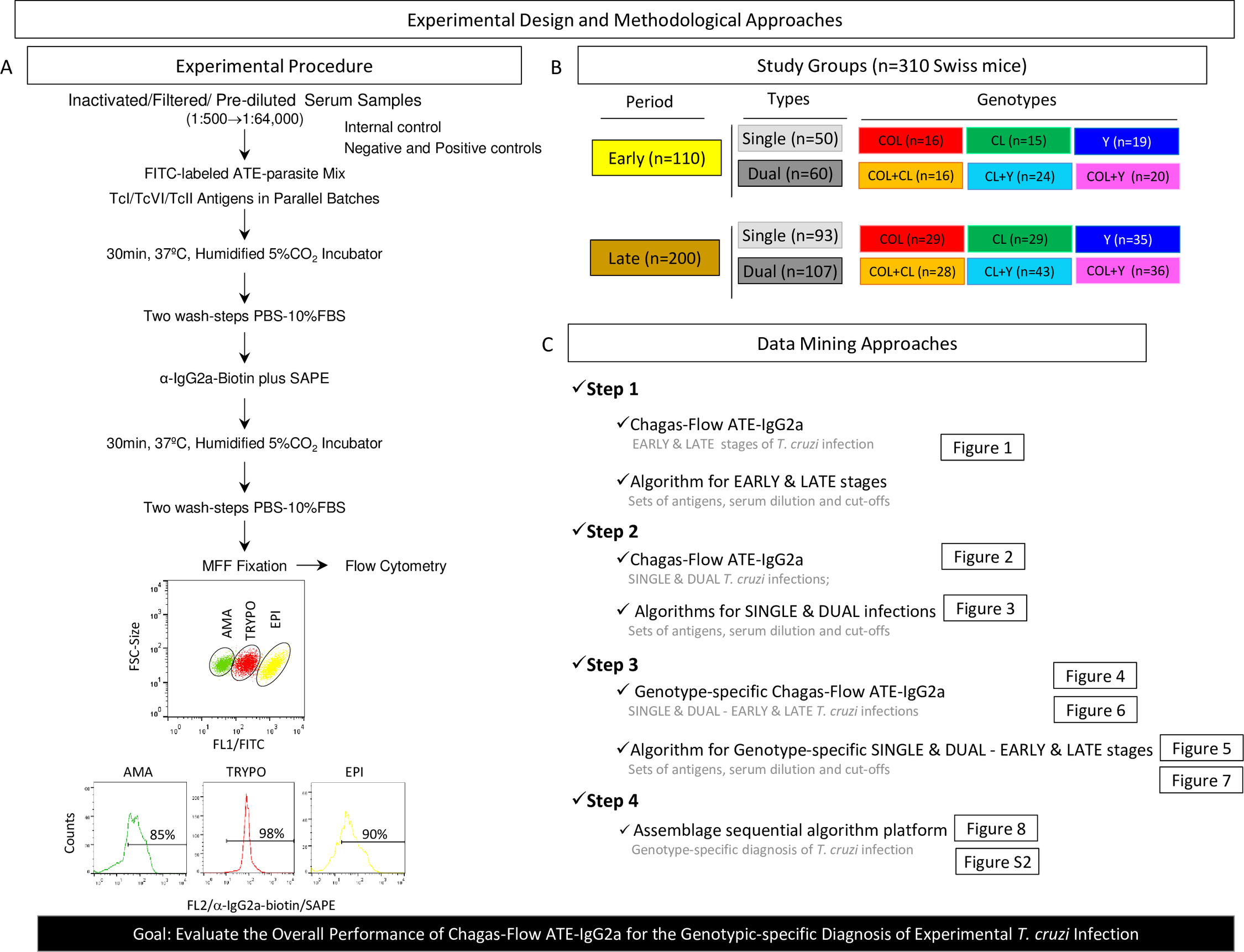

Supplement: S1 Fig — (A) Schematic flow chart overview of the experimental procedure Chagas-Flow ATE-IgG2a. Representative gating strategies to select the target antigens (amastigote-AMA = green, trypomastigote-TRYPO = red and epimastigote-EPI = yellow) and the histograms employed to quantify the percentage of positive fluorescent parasites (PPFP). (B) The compendium of the study population comprised of Swiss mice, categorized into subgroups referred as: early (yellow), late (brown), single (light gray), dual (dark gray), TcI/COL (red), TcVI/CL (green), TcII/Y (dark blue), TcI/COL+TcVI/CL (orange), TcVI/CL+TcII/Y (light blue) and TcI/COL+TcII/Y (pink). (C) Data mining approaches was achieved through the steps: Step 1 (algorithm for early & late stages); Step 2 (algorithms for single & dual infections); Step 3 (algorithm for genotypic-specific single & dual—early & late stages); Step 4 (assemblage sequential algorithm platform). (TIF) [file pntd.0006140.s001.tif]

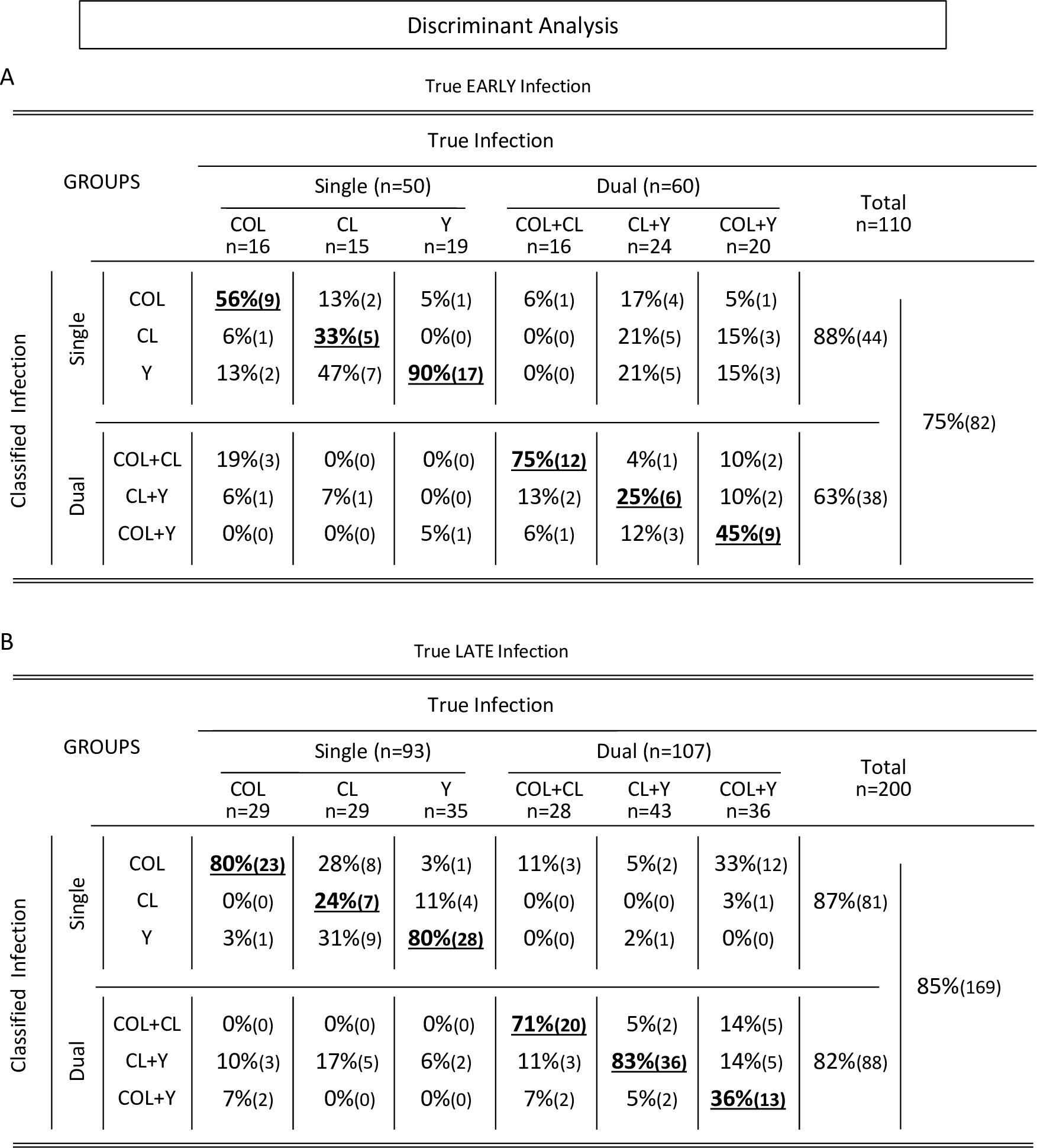

Supplement: S2 Fig — Discriminant analyses of combined Chagas-Flow ATE-IgG2a for genotype-specific diagnosis at (A) early and (B) late stages of T. cruzi single infection (COL, CL and Y) and dual infection (COL+CL, CL+Y and COL+Y). The global accuracy is provided in the Figure. (TIF) [file pntd.0006140.s002.tif]

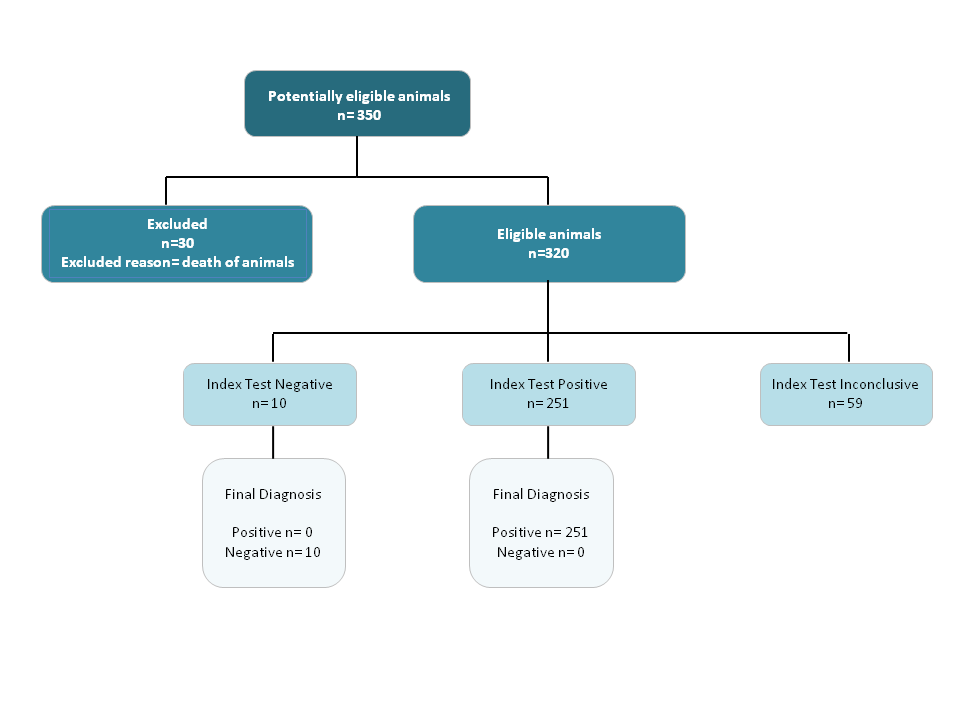

Supplement: S3 Fig — (TIF) [file pntd.0006140.s003.tif]
